# Asymmetric valuation of gains and losses in effort-based decision making

**DOI:** 10.1371/journal.pone.0223268

**Published:** 2019-10-15

**Authors:** Megan K. O’Brien, Alaa A. Ahmed

**Affiliations:** Department of Integrative Physiology, University of Colorado Boulder, Boulder, CO, United States of America; Brain and Spine Institute (ICM), FRANCE

## Abstract

Our decisions are often swayed by a desire to avoid losses over a desire to acquire gains. While loss aversion has been confirmed for decisions about money or commodities, it is unclear how individuals generally value gains relative to losses in effort-based decisions. For example, do individuals avoid greater work more than they seek out less work? We examined this question in the context of physical effort, using an arm-reaching task in which decreased effort was framed as a gain and increased effort was framed as a loss. Subjects performed reaching movements against different levels of resistance that increased or decreased the effort demands of the reaches. They then chose to accept or reject various lotteries, each with a possibility of performing less effortful reaches and a possibility of performing more effortful reaches, compared to the certain outcome of performing reaches against a fixed reference level of effort. Subjects avoided higher effort conditions more than they sought lower effort conditions, demonstrating asymmetric valuation of gains and losses. Using prospect theory, we explored various model formulations to determine subject-specific valuation of effort in these mixed gambles. A nonlinear model of effort valuation demonstrating increasing sensitivity to absolute effort best described the effort lottery choices. In contrast to the loss-aversion observed in financial decisions, there was no evidence of loss aversion in effort-based decisions. Rather, we observed moderate relief-seeking behavior. This model confirms that gains and losses are valued asymmetrically. This is due to the combined effects of increasing sensitivity to absolute effort and moderate relief-seeking, leading to a net effect of greater avoidance of higher effort. Asymmetric valuation was magnified on a later day of testing. In contrast, subjects were loss-averse in a comparable financial task. We suggest that consideration of nonlinear effort valuation can inform future studies of sensorimotor control and exercise motivation.

## Introduction

Loss aversion is a well-established phenomenon in human behavior. Our desire to avoid negative outcomes (losses) often surpasses our desire to acquire positive outcomes (gains), notably affecting our financial and commodity-based decisions [1]. For instance, imagine you have the opportunity to play a lottery with a 50:50 chance of winning $100 and losing $100. A rational decision maker would have no preference between playing the lottery (with an expected value of $0) and refusing to play it (which would also have an expected value of $0). However, most people would reject the opportunity to play this lottery, and would choose not to play unless the potential gain is larger than the potential loss. Prospect theory [2, 3]–a landmark descriptive model of decision making–explains such risk-averse behavior using the concept of loss aversion, wherein people are more upset by a loss than they are pleased by an equivalent gain. Loss aversion is represented by an asymmetric value function, meaning the subjective valuation of outcomes is steeper for losses than for gains (“losses loom larger than gains”). While loss aversion is a fixture in economic decision making, it is unknown whether this behavior occurs in other domains, such as effort. Decisions must be acted upon, and this action invariably involves physical effort, but do individuals value effort symmetrically? To our knowledge, there has been no explicit examination of the relative valuation between effort-based gains and losses.

It has long been assumed that effort influences our choices, generally such that we make choices that minimize effort expenditure. This idea is indirectly encoded in Hull’s *law of less work* [4], stating that organisms choosing between equally reinforced actions will learn to select actions that require less energy expenditure. Behavioral studies have confirmed humans and other animals consider different effort costs in their decision-making processes [5]. Indeed, they tend to select low-effort movement strategies: reaching in directions that involve moving less mass [6, 7], walking along paths that require the fewest number of steps [8], preferring to move an object over shorter distances using less rotation [9], and exploiting motor redundancy in unfamiliar tasks [10]. During arm-reaching movements, increasing effort reduces the frequency of changes of mind after beginning a movement toward an uncertain target, suggesting that the criteria to correct a movement are dependent on energetic costs [11]. Effort reduction and motor learning also appear to be linked, as there is a distinct reduction in metabolic power during the learning of novel arm reaching dynamics [12–14]. There is also evidence that people avoid cognitive effort, consistently avoiding outcomes with greater mental demands [15].

Not only does effort affect our choice behavior, but these costs are also susceptible to neuroeconomic phenomena that have been observed in decisions involving other commodities. Effort, like time, has been shown to discount rewards [5, 16–20], and people exhibit risk-sensitivity in tasks involving physical effort [21] and cognitive effort [20]. As such, other heuristics and biases that characterize our decision making processes may apply to effort as well.

We applied the prospect theory framework to examine decision making in an effort-based task, specifically investigating how the relative valuation of effort gains and losses affects movement decisions. Determining the relationship between gains and losses in an effort domain would allow us to more fully capture, predict, and motivate movement behavior. If overall energy expenditure is a cost, we might consider reductions in effort (i.e. effort relief) to be the gains, whereas additions in effort would be cast as losses. Thus, loss aversion would manifest as a stronger aversion to increased effort than an inclination toward decreased effort. We tested the hypothesis that healthy young adults exhibit loss aversion in an effortful reaching movement. We expected that loss aversion in this physical effort task would have a similar magnitude to that seen in classic financial tasks (wherein losses loom larger than gains). Alternatively, individuals could exhibit symmetric valuation of the effort-based gains and losses, or asymmetric valuation due to nonlinear representations of absolute effort.

## Results

**Fig 1:**
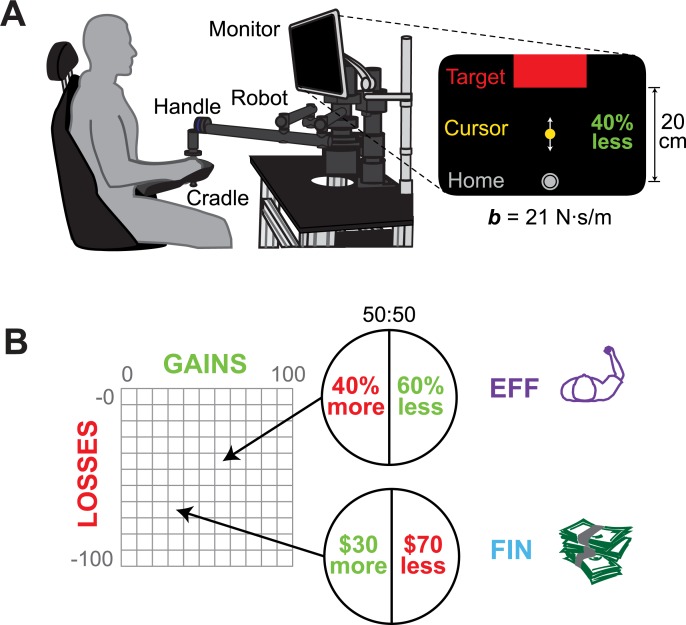
Experimental setup. (**A**) Subjects were trained in planar reaching movements using a robotic arm. Effort was varied using a viscous force field, designated as percentages more or less than an intermediate “reference” effort. (**B**) During testing, subjects were shown 50:50 gain-loss lotteries with more or less effort (EFF) and with more or less money (FIN).

We designed an effort-based task in which subjects performed out-and-back arm-reaching against a viscous force. We manipulated the level of resistance encountered during reaching by altering the viscous damping coefficient *b* of the force, thereby making the reach more or less effortful (Fig 1A). After training to reach at a range of resistances (0 ≤ *b* ≤ 70 N⋅s/m), subjects made choices between a sure bet and a lottery relating to the effort task (EFF; Fig 1B). In rejecting the lottery (thereby choosing the sure bet), subjects would have to reach against a moderate “reference” resistance (*b* = 35 N⋅s/m; defined as 0% gain/loss). In accepting the lottery, subjects would have a 50:50 possibility of reaching against a lower resistance (gain) or a higher resistance. After making an initial choice to accept or reject a lottery, subjects indicated whether this was a strong or weak choice. Gains were presented as a percentage decrease in effort, from 0–100% in increments of 10%, which corresponded to resistances 35 ≤ *b* ≤ 0 N⋅s/m in increments of 7 N⋅s/m. Losses were presented as a percentage increase in effort, from 0–100% in increments of 10%, which corresponded to resistances 35 ≤ *b* ≤ 70 N⋅s/m in increments of 7 N⋅s/m.

Subjects chose between the sure bet and the lottery for various gains and losses, and one choice was realized during 10 minutes of reaching at the end of the experiment. On a second day of testing performed at least one week later, subjects made choices in an analogous financial task (FIN; Fig 1B), in which they chose between a sure monetary bet and a lottery with various monetary gains and losses ranging from $0-$100. To determine the consistency of effort-based choices, subjects also repeated the effort task on this second day of testing (EFF2).

We quantified loss aversion by fitting subjects’ decisions to a model of choice based on prospect theory. A curvilinear value function with 3 free parameters was used to describe the subjective value of an outcome *X* (SV = *X*^*α*^, for *X* > 0; SV = −*λ*(−*X*)^*α*^ for *X* < 0), where the value sensitivity parameter *α* describes the curvature of the function and the loss-aversion coefficient *λ* describes how losses are valued relative to gains. An additional parameter, *μ*, describes choice stochasticity (see Materials and Methods). Two nested choice models were also considered, to test for the absence of value sensitivity (*α* = 1) and the subsequent absence of loss aversion (*α* = *λ* = 1). These choice models were tested for both linear and nonlinear (quadratic) representations of effort. These were compared against an additional curvilinear value model assuming the absence of a reference point (all effort viewed as a loss). Essentially, this model fits an exponent, *γ*, on the absolute effort function: *X* = *b*^*γ*^. Lastly, we tested a hybrid model combining a traditional prospect theory model with reference-dependent loss aversion, *λ*, that assumes an underlying linear absolute effort valuation function (i.e. *X* = Δ*b*), with a model that allows for nonlinearities in the absolute encoding function, *X* = *Δ*(*b*^*γ*^). This hybrid model was tested to determine the extent to which asymmetric valuation could be explained by a combination of nonlinear absolute effort valuation, reflected in the exponent *γ*, and reference-dependent loss-aversion, *λ*. Model parameters were fit using maximum likelihood estimation, then compared between the effort-based choice task and a similar financial choice task.

### Movement behavior and lottery choices

Average velocities across training conditions were computed to confirm that subjects experienced the desired forces in this velocity-dependent field. Indeed, subjects maintained similar velocities across resistance conditions (Table 1), and the average force they encountered scaled linearly with resistance (R^2^ = 0.9997, F = 2.66x10^4^, p<0.001). This verifies that the effort gains and losses used in the model are sufficient since subjects generally experienced the designated effort conditions.

**Table 1:** Average (±SD) movement velocities and durations for each effort condition with damping coefficient *b*.

Condition (N⋅s/m)	0	7	14	21	28	35	42	49	56	63	70
**Average velocity (m/s)**	0.35 (0.03)	0.35 (0.03)	0.36 (0.03)	0.35 (0.03)	0.35 (0.03)	0.34 (0.03)	0.35 (0.03)	0.35 (0.03)	0.35 (0.02)	0.35 (0.03)	0.34 (0.03)
**Peak velocity** **(m/s)**	0.58 (0.03)	0.57 (0.03)	0.58 (0.02)	0.57 (0.03)	0.56 (0.02)	0.56 (0.03)	0.55 (0.03)	0.55 (0.03)	0.55 (0.02)	0.54 (0.03)	0.54 (0.03)
**Duration (s)**	0.55 (0.06)	0.55 (0.05)	0.54 (0.06)	0.54 (0.06)	0.54 (0.07)	0.58 (0.05)	0.55 (0.05)	0.55 (0.05)	0.55 (0.05)	0.55 (0.06)	0.56 (0.06)

**Fig 2:**
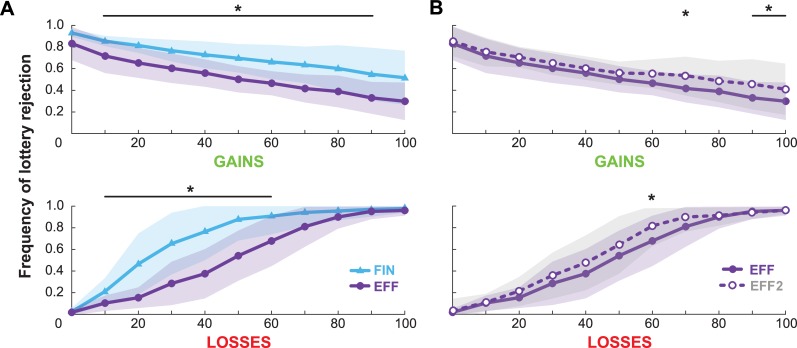
Frequency of rejecting lotteries. Comparison of mean and standard deviation of lottery rejection for (**A**) EFF and FIN tasks. and (**B**) EFF and EFF2 tasks. In all tasks, the frequency of rejection decreases with larger gains and increases with larger losses. Asterisks (*) span across gain/loss values for which there is a significant difference between the two compared tasks.

In the EFF, EFF2, and FIN lottery tasks, Mann-Kendall tests reveal that the average number of rejected lotteries decreased monotonically with increasing gains (*N* = 11, p<0.030) and increased with increasing losses (*N* = 11, p<0.004), confirming that subjects valued increases in effort as aversive and reductions in effort as desirable (Fig 2). Lotteries were rejected more frequently in FIN than EFF for gains between 10 and 90 and for losses between 10 and 60 (paired t-tests; *df* = 10, p’s<0.0045). Lotteries were rejected more frequently in EFF2 than EFF for gains of 70, 90, and 100, as well as for losses of 60 (paired t-tests; *df* = 10, p’s<0.0045).

**Fig 3:**
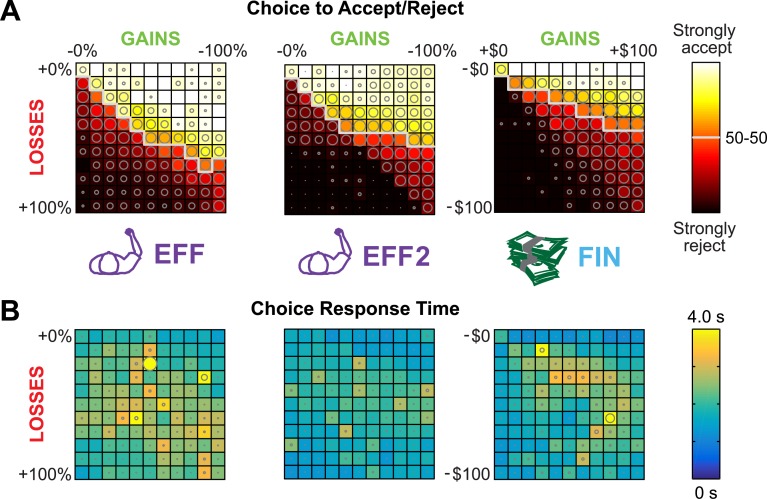
Example decision matrices. (**A**) Average frequency of accepting and rejecting lotteries across gains and losses and (**B**) average response times to the first accept/reject decision for the initial effort task (left), the repeated effort task (middle), and an equivalent financial task (right). Sizes of the overlaid circles represent the within-lottery standard deviation, plotted relative to the maximum SD across tasks (for frequency, SD_max_ = 0.51 from FIN; for response time, SD_max_ = 23.1 s from EFF). Gray line in frequency matrices denotes the 50% accept/reject boundary, above which subjects accepted more often and below which they rejected more often.

Average decision matrices in each task are shown in Fig 3A, directly illustrating a higher rejection rate for the financial lotteries than the effort lotteries, as well as a higher rejection rate for the repeated effort task than the initial effort task. Average response times for each initial choice (to accept or reject the lottery) are shown in Fig 3B. Response times *t* were significantly higher in EFF than in FIN (paired t-test; *t*(17) = 5.07, p<0.001), with across-subjects mean (±SD) *t*_EFF_ = 2164 (±364) and *t*_FIN_ = 1730 (±447) ms. Response times were significantly lower in EFF2 than in EFF (paired t-test; *t*(17) = 4.55, p = 0.023), with *t*_EFF2_ = 1706 (±361) ms.

### Evidence for asymmetric valuation of gains and losses

Parameter fits for the EFF models are given in Table 2. The 3-parameter hybrid model of nonlinear effort was best able to explain subject choices in the effort task, providing the highest protected exceedance probability of 0.99, as determined from group-level Bayesian model selection (BMS). The parameter fits were not normally distributed (Shapiro-Wilk test: p<0.03), so we report the median and interquartile range (IQR) and compare values using Wilcoxon signed-rank tests. For the EFF task, median *λ*_EFF_ = 0.71 (IQR: 0.14–0.99), and this value was significantly less than 1.0 (p = 0.006). Median *γ*_EFF_ = 1.61 (IQR: 1.21–4.21), and this was also significantly different from 1.0 (p = 0.0003). A model confusion analysis confirmed the winning hybrid model was fully identifiable from the alternative models, indicating reliability in the model selection process.

**Table 2:** Median and IQR parameter fits for alternative value functions and effort models in the EFF task, with posterior model frequencies and protected exceedance probabilities (pxp). The winning model is the loss aversion and nonlinear value encoding model, with Bayesian omnibus risk *P*_*0*_ = 4.6e-7.

Model	# param	Value function: gains (*X*>0)	Value function: losses (*X*<0)	Gain/loss choices, *X*	*λ*_EFF_	*α*_EFF_	*γ*_EFF_	*μ*_EFF_	Posterior freq	pxp
Symmetric sensitivity	3	*X*^*α*^	−*λ*(−*X*)^*α*^	*X* = Δ*b*	1.17* [0.98, 1.31]	0.84 [0.67, 1.09]	N/A	1.08 [0.37, 2.25]	0.073	2.26x10^-4^
				*X* = Δ(*b*^2^)	0.64* [0.48, 0.80]	0.76 [0.52, 1.14]	N/A	0.11 [0.004, 0.63]	0.066	1.13x10^-4^
No sensitivity	2	*X*	−*λ*(−*X*)	*X* = Δ*b*	1.20* [0.95, 1.30]	N/A	N/A	0.52 [0.33, 0.69]	0.039	2.49x10^-5^
				*X* = *Δ*(*b*^2^)	0.55* [0.43, 0.62]	N/A	N/A	0.012 [0.008, 0.014]	0.037	2.29x10^-5^
No loss aversion	1	*X*	−*X*	*X* = Δ*b*	N/A	N/A	N/A	0.40 [0.25, 0.62]	0.038	2.19x10^-5^
				*X* = *Δ*(*b*^2^)	N/A	N/A	N/A	0.003 [0.002, 0.004]	0.036	2.09x10^-5^
Zero-effort reference	2	N/A	−*X*	*X* = *b*^*γ*^	N/A	N/A	1.24* [1.00, 1.33]	0.21 [0.10, 0.67]	0.041	4.69x10^-5^
Loss aversion + encoding	3	*X*	−*λ*(−*X*)	*X* = Δ(*b*^*γ*^)	0.71* [0.14, 0.99]	N/A	1.61* [1.21, 4.21]	0.064 [0.00, 0.34]	0.67	0.999

* Significantly different from 1.0 (Wilcoxon signed-rank test).

In this nonlinear model of effort encoding, interpretation of the parameter fits can be complex. First, we start with a baseline perceived effort that scales nonlinearly, specifically to the power *γ*, with damping coefficient *b*. Since *γ* is greater than one, increases in effort relative to the reference (losses) are already valued more steeply than decreases in effort (gains) when viewed in terms of the damping coefficient, *b*. This alone could explain the asymmetric valuation observed in the subjects’ decisions. However, the parameter *λ* further modifies the gain-loss symmetry and value sensitivity relative to this nonlinear representation. The loss aversion parameter, *λ*, was less than one, indicating a moderate degree of effort-relief, rather than the effort (loss) aversion. Importantly, this effort relief is relative to the asymmetry already present in the nonlinear effort valuation. Here these two parameters act in different directions, whereby *λ* acts to counteract the increasing absolute effort sensitivity already present in the nonlinear valuation. However, the increasing absolute effort sensitivity is stronger and the net effect is behavior where increases in effort are valued more steeply than equivalent reductions in effort.

**Fig 4:**
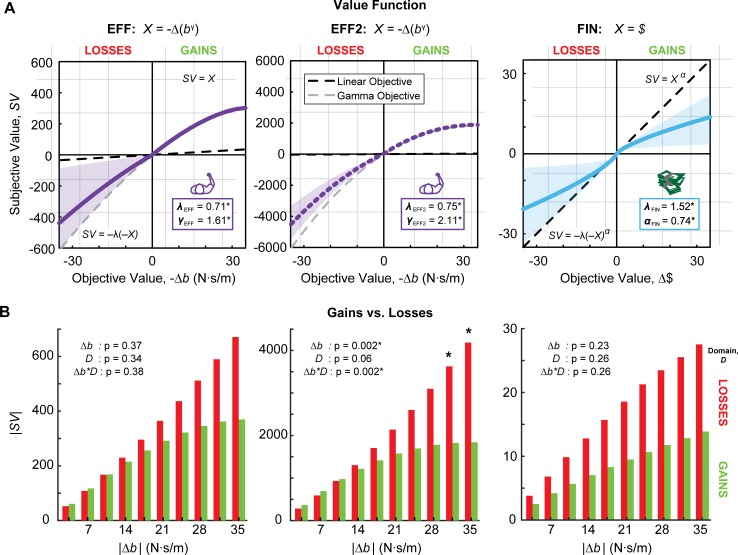
Model fits: Effort initial vs. repeated. (**A**) Subjective value functions of in the EFF, EFF2, and FIN tasks, with median parameter fits from the respective winning models in each task. Median and 95% confidence intervals resulting from *α* and *λ* are shown. The gray dashed line represents objective valuation for an effort model with median *γ* value for that task (showing baseline asymmetry in valuation of gains and losses, to determine the relative contribution of *λ* in nonlinear effort encoding). The black dashed line represents *SV = X* for a linear effort model, for comparison. (**B**) Median subjective valuation for each loss/gain condition over all subjects. Results of two-way repeated-measures ANOVA reveal main effect of condition and an interaction effect with gain/loss domain in the EFF2 task. Asterisks (*) from post-hoc testing denote significant difference between gain and loss valuation for that condition.

The subjective valuation curve from this winning model in the EFF task is illustrated in Fig 4, along with the distribution of gain and loss valuations across subjects in each effort condition. To estimate the relative valuation of gains and losses, the subjective value of each loss condition (e.g. Δ*b* = 7 N⋅s/m) can be divided by the subjective value of the equivalent gain condition (e.g. Δ*b* = -7 N⋅s/m). The resulting ratio was averaged across conditions for each subject. The median loss/gain valuation was 0.99 (IQR: 0.91–1.24). A two-way repeated measures ANOVA examined the within-subjects effects of domain (gain vs. loss) and effort condition (|Δ*b*|; absolute change in resistance relative to the reference) on the subjective valuation of effort, as well as their interaction. There was no effect of domain (p = 0.37) or condition (p = 0.34), nor an interaction effect (p = 0.38), suggesting that there were no detectable differences in the valuation of gains and losses in the EFF task.

The hybrid 3-parameter model of nonlinear effort was also the winning model for the repeated effort task, EFF2, with a protected exceedance probability of 0.96 (Table 3). The median model estimates from this task were *λ*_EFF2_ = 0.75 (IQR: 0.55–0.90; p = 0.016) and *γ*_EFF2_ = 2.11 (IQR: 1.20–2.86; p = 0.0005). Wilcoxon signed-rank tests reveal that loss aversion coefficients are not different between the two days (p = 0.88), but value sensitivity *γ* is greater on the second day of testing(p = 0.024). The subjective valuation curve and median gain and loss valuations for EFF2 is illustrated in Fig 4. The median ratio of loss/gain valuation was 1.28 (IQR: 1.10–1.76) in the EFF2 task, confirming that losses were valued more strongly than gains in the repeated effort task. There was no effect of domain (p = 0.06), whereas there was a significant effect of condition (p = 0.002) and an interaction between condition and domain (p = 0.002). Post-hoc comparisons, corrected using Tukey’s Honestly Significant Difference, revealed that losses are valued steeper than gains when the difference in effort from the reference is largest: |Δ*b|* = 31.5 N⋅s/m (p = 0.041) and 35 N⋅s/m (p<0.001).

**Table 3:** Median and IQR parameter fits for alternative value functions and effort models in the EFF2 task, with posterior model frequencies and protected exceedance probabilities (pxp). The winning model is the loss aversion and nonlinear value encoding model, with Bayesian omnibus risk *P*_*0*_ = 3.4e-5.

Model	# param	Value function: gains (*X*>0)	Value function: losses (*X*<0)	Gain/loss choices, *X*	*λ*_EFF_	*α*_EFF_	*γ*_EFF_	*μ*_EFF_	Posterior freq	pxp
Symmetric sensitivity	3	*X*^*α*^	−*λ*(−*X*)^*α*^	*X* = Δ*b*	1.36* [1.14, 2.15]	0.97 [0.67, 1.23]	N/A	0.78 [0.24, 2.57]	0.076	1.77x10^-3^
				*X* = Δ(*b*^2^)	0.89 [0.61, 0.97]	0.65 [0.31, 0.96]	N/A	0.22 [0.016, 2.58]	0.15	3.47x10^-2^
No sensitivity	2	*X*	−*λ*(−*X*)	*X* = Δ*b*	1.53* [1.17, 1.77]	N/A	N/A	0.49 [0.33, 0.68]	0.041	4.16x10^-4^
				*X* = Δ(*b*^2^)	0.73* [0.52, 0.88]	N/A	N/A	0.012 [0.008, 0.017]	0.042	4.86x10^-4^
No loss aversion	1	*X*	−*X*	*X* = Δ*b*	N/A	N/A	N/A	0.30 [0.27, 0.59]	0.039	3.93x10^-4^
				*X* = Δ(*b*^2^)	N/A	N/A	N/A	0.005 [0.003, 0.009]	0.039	3.50x10^-4^
Zero-effort reference	2	N/A	−*X*	*X* = *b*^*γ*^	N/A	N/A	1.50* [1.12, 1.80]	0.06 [0.03, 0.29]	0.042	5.02x10^-4^
Loss aversion + encoding	3	*X*	−*λ*(−*X*)	*X* = Δ(*b*^*γ*^)	0.75* [0.55, 0.90]	N/A	2.11* [1.20, 2.86]	0.01 [0.001, 0.65]	0.58	0.961

* Significantly different from 1.0 (Wilcoxon signed-rank test).

### Comparison with loss aversion in financial task

Parameter fits for the FIN models are given in Table 4. The conventional prospect theory 3-parameter model with loss-aversion and symmetric sensitivity was best able to explain subject choices in the FIN task, with a protected exceedance probability of 0.96. Because this was a linear model of monetary lotteries, the impact of parameters *λ* and *α* on the subjective valuation curve is more straightforward. Subjects were loss-averse in the financial task, with *λ*_FIN_ = 1.52 (IQR: 1.13–2.37; p<0.001), and this value was significantly greater than *λ*_EFF_ (p<0.001). They also demonstrated diminishing sensitivity to financial values, *α*_FIN_ = 0.74 (IQR: 0.35–0.87; p = 0.008). Median parameter fits for *μ* were *μ*_EFF_ = 0.064 (IQR: 6x10^-6^–0.338) and *μ*_FIN_ = 1.24 (IQR: 0.49–10.85). The two different winning model formulations across tasks prevents any further meaningful comparison of the parameters. The subjective valuation curve and comparison of median losses and gains for FIN are given in Fig 4. Similar to EFF, there was no effect of domain (p = 0.23) or condition (p = 0.26), nor an interaction effect (p = 0.23) for the FIN task.

**Table 4:** Median and IQR parameter fits for alternative value functions in the FIN task, with posterior model frequencies and protected exceedance probabilities (pxp). The winning model is the symmetric sensitivity model, with Bayesian omnibus risk *P*_*0*_ = 0.0088.

Model	# param	Value function: gains (*X*>0)	Value function: losses (*X*<0)	Gain/loss choices, *X*	*λ*_FIN_	*α*_FIN_	*γ*_FIN_	*μ*_FIN_	Posterior freq	pxp
Symmetric sensitivity	3	*X*^*α*^	−*λ*(−*X*)^*α*^	*X* = $ [−100:10:100]	1.52* [1.13, 2.37]	0.74* [0.35, 0.87]	N/A	1.24 [0.49, 10.85]	0.54	0.96
No sensitivity	2	*X*	−*λ*(−*X*)	*X* = $ [−100:10:100]	1.87* [1.22, 3.01]	N/A	N/A	0.18 [0.16, 0.39]	0.18	1.51x10^-2^
No loss aversion	1	*X*	−*X*	*X* = $ [−100:10:100]	N/A	N/A	N/A	0.10 [0.05, 0.11]	0.044	1.73x10^-3^
Large-money reference	2	*X*	N/A	*X* = $^*γ*^ [0:10:200]	N/A	N/A	0.002* [0.00, 0.71]	1.54x10^4^ [1.98, 2.55x10^4^]	0.044	1.72x10^-3^
Loss aversion + encoding	3	*X*	−*λ*(−*X*)	*X* = $^*γ*^ [−100:10:100]	0.901 [0.74, 1.10]	N/A	0.001* [0.001, 0.70]	2.02x10^4^ [2.24, 3.5x10^4^]	0.20	2.05x10^-2^

* Significantly different from 1.0 (Wilcoxon signed-rank test).

**Fig 5:**
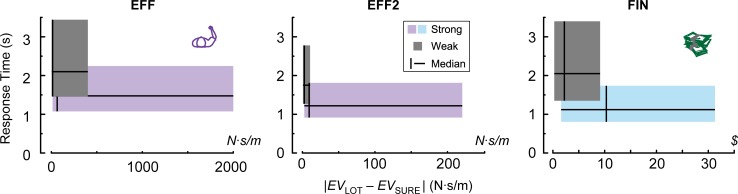
Response times for strong and weak decisions. Distribution of response times for the initial decision to accept or reject a lottery and the differences in expected value (EV) between the lottery and the sure bet. Median values are shown as black lines, and the IQR is shown as a box. Weak decisions were made after a longer response time and occurred when difference in EV was small.

A contrast between the strong and weak accept/reject data is presented in Fig 5, illustrating how the difference in subjective value between the lottery and the sure bet affected the strength of a decision and the response time. The expected value (EV) of each lottery and of the sure bet was computed as the sum of subjective values for the gains and losses multiplied by their respective probabilities. Subjective values were derived from each subject’s estimated *λ* and *α* parameters. As the difference in subjective values increased, subjects responded more quickly and more strongly, Conversely, weak responses were given when the difference in EV between the lottery and the sure bet was small, and subjects took a longer time to make the initial accept or reject decision. Median response times for weak choices were 2100 ms (IQR: 1460–3440) in EFF, 1750 ms (IQR: 1270–2778) in EFF2, and 2050 ms (IQR: 1350–3400) in FIN. Median response times for strong choices were 1475 ms (IQR: 1075–2245) in EFF, 1220 ms (IQR: 915–1810) in EFF2 and 1120 ms (IQR: 805–1735) in FIN. The larger range of differences in EV in the EFF task results from the increased prevalence of subjects with *γ* > 1 in this task, which inflates the subjective value of outcomes.

### Consistency of parameter estimates between tasks

**Fig 6:**
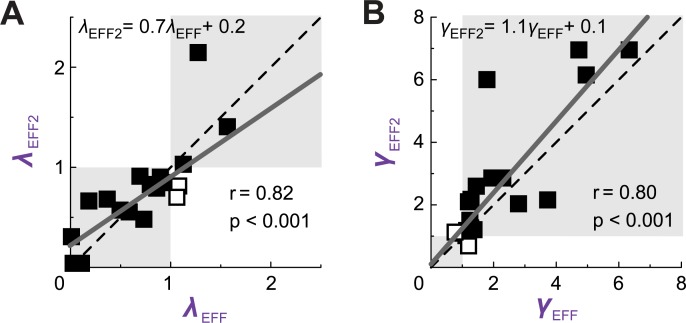
Consistency of parameters. For each subject, comparison of EFF and EFF2 parameter fits for (**A**) loss aversion and (**B**) value sensitivity. Closed squares (points in the shaded region) indicate similar directionality in parameter estimates between the two tasks, whereas open squares indicate opposing directionality. Linear regression lines are also shown for these significant correlations.

Estimates of *λ* were strongly correlated between EFF and EFF2 sessions (r = 0.82, p<0.001; Fig 6A), as were estimates of *γ* (r = 0.80, p<0.001; Fig 6B). Of the 18 subjects who participated in the second testing session (in which they repeated the effort lotteries), 16 showed consistent directionality in loss-aversion coefficients, and 16 showed consistent directionality in value sensitivity.

### Evidence for nonlinear representation effort encoding

One assumption about the arm-reaching task is that effort increases linearly with increasing resistance, and that subjects perceive these differences to be linear and uniform (i.e. increasing resistance by 7 N⋅s/m results in and feels like a 10% increase in effort, as per the mapping between *b* and *b*_shown_). This assumption may be reasonable when considering that the objective representation of effort is the metabolic cost required to perform the task, which can be approximated as the sum of forces [5, 22, 23].

**Fig 7:**
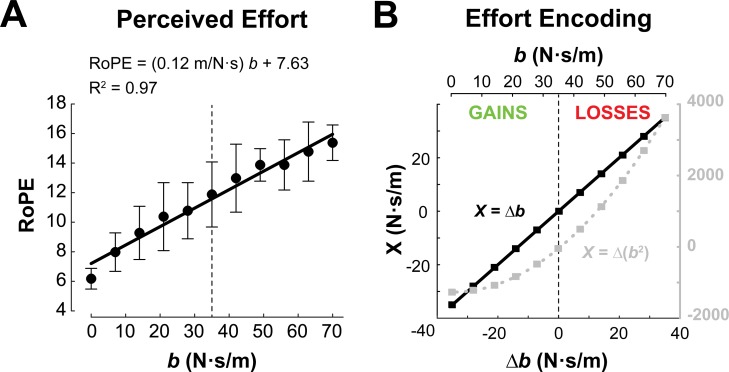
Representations of effort. (**A**) Mean and standard deviation of perceived exertion (RoPE; from Borg’s Ratings of Perceived Exertion scale), plotted as a function of damping coefficient *b*. (**B**) Effort lotteries were encoded as either a linear (black, left axis) or quadratic (gray, right axis) function of the changes in damping coefficient (relative to reference). Dashed lines denote the reference effort condition (35 N⋅s/m) separating gains and losses.

During training, subjects were asked to report their rating of perceived exertion (RoPE). RoPE increased with resistance and can be approximated as a linear function (Fig 7A, R^2^ = 0.97, F = 283.73, p<0.001), supporting the idea that the linear gain and loss conditions are appropriate inputs to the prospect theory framework (Fig 7B).

However, there is ongoing debate about how the brain encodes effort. Various theories of optimal motor control assume quadratic effort costs [24–27]. Furthermore, recent work in decision making has demonstrated the presence of effort discounting and nonlinear sensitivity to increasing effort [19, 28, 29], and subjective effort costs are not exclusively related to energy expenditure [30, 31]. As such, asymmetric valuation of effort gains and losses may arise from nonlinear effort perception rather than loss aversion, which is why a quadratic effort representation was also explored in our model space (Fig 7B). To better disassociate the effects of nonlinear absolute effort encoding and reference-dependent loss aversion, we considered a hybrid model in which both parameters (absolute effort sensitivity and reference-dependent loss aversion) to subject behavior. Our results suggest that subjects maintain a nonlinear absolute effort sensitivity, and this leads to asymmetric valuation of increases in effort compared to equivalent reductions in effort.

## Discussion

We investigated the question of how increases in physical effort (losses) are subjectively valued relative to decreases in physical effort (gains). Subjects were trained to perform reaching movements against different levels of resistance, and they chose to accept or reject related effort-based lotteries. We found that a nonlinear model of effort with increasing effort sensitivity and no loss aversion provided the best fit to subject choices. More specifically, the model revealed moderate relief seeking tendencies, which were cancelled out by the increasing sensitivity, leading to a net effect of greater effort avoidance. This model outperformed alternative models with linear effort encoding or symmetric valuations of gains and losses. It also outperformed a 2-parameter zero-reference model with no loss aversion coefficient.

We observed that increasing effort was rejected more than decreasing effort was accepted (Fig 3A). Our model analysis reveals that such behavior stems from a combination of nonlinear effort representation, and loss aversion coefficient that is less than 1 (relief-seeking). In the winning model of nonlinear effort encoding, higher effort conditions are valued more steeply than lower effort conditions. This aligns with a recent study by Morel et al. [31], in which a quadratic effort model was best able to explain subject choices in an effortful reaches of varying resistance, amplitude, and duration. However, our results here do not align with a quadratic encoding, but rather a weaker nonlinearity. A model assuming an underlying quadratic encoding, performed significantly worse. In contrast, an equivalent financial task tested under the same linear model set showed that a traditional 3-parameter loss aversion model outperformed the alternatives, in which subjects valued losses more strongly than gains by a median factor of 1.52. This agrees with previous findings of loss aversion in the economic domain [2, 3, 32, 33]. Ultimately, the gain/loss valuation in the effort and financial domains do not appear to be linked, though asymmetry was seen in both.

Asymmetric effort valuation emerged more strongly on the second day of testing, approximately one week after the initial testing session, wherein subjects demonstrated greater value sensitivity, and the effort value function was steeper for losses than for gains by a median factor of 1.28. A possible explanation for this behavior lies in the difference between anticipating outcomes and experiencing outcomes. Experience with losses may attenuate loss aversion [34, 35] because repeated exposure to losses teaches people that the negative outcome is not as bad as they predicted. In our experiment, subjects undergo a lengthy training procedure prior to the initial testing session. Reduced training during the second day of testing may have elevated effort aversion, with subjects relying more on forecasting than direct experience. Alternatively, the experience gathered when playing out a lottery at the end of the initial session (requiring 10 minutes of arm-reaching against some resistance) induced effort aversion during the second session. Subjects may have been more sensitive to effort during the second session after having experienced the lottery play-out. Behaviorally, this manifested as accepting fewer high-gain lotteries and rejecting more middle-loss lotteries (Figs 2B and 3A).

In their formulation of prospect theory, Kahneman and Tversky [36] introduce the reference-dependent property of loss aversion, wherein gains and losses are all measured relative to a reference point. The authors note that the formulation of risky prospects and the expectations of the decision maker can affect the location of this reference. Current models of sensorimotor control often incorporate effort as a cost to be minimized but do not account for the possible existence of a reference point. Importantly in our study, subjects would always have to exert some amount of effort regardless of their lottery decisions, imparted through the 10 minutes of reaching performed at the end of the experiment. An implicit assumption of these models is that some amount of effort must be exerted, and an optimal movement minimizes those effort costs. We established a reference point in the form of an intermediate level of resistance (*b* = 35 N⋅s/m, presented as 0% more/less effort). Effort-based gains and losses were presented as decreases and increases from the reference. A choice model fitting a curvilinear value function and loss aversion relative to this reference fit the data better than an alternative model with a zero-effort reference point. We have thusly demonstrated that a reference point in effort can be externally imposed, confirming that prospect formulation can affect its location. The reference point may change for different arrangements of risky choices and for other movement tasks.

Our findings may explain behavioral studies in which individuals do not appear to prioritize effort minimization. For instance, Kistemaker et al. [37] designed a force field environment for arm reaching so that minimal energy trajectories deviated appreciably from trajectories in a null field. After practicing reaches along the minimal energy path, subjects did not alter their movements from those performed in the null field, thereby using more energy than needed to complete the task. One interpretation of this experiment is that minimization of effort is of lesser importance in determining movement behavior. On the other hand, our findings suggest that the central nervous system possesses a more complicated representation of effort costs, in which effort-based gains and losses are valued asymmetrically. Indeed, if increases in effort were valued more strongly than decreases in effort, subjects would not necessarily seek the lower effort paths. Additionally, because of the seemingly dynamic nature of the reference point in effort-based valuation, the force field introduced in that experiment may have altered subjects’ perceptions of a reference effort. They would thus prefer avoiding more effortful movements (above that reference) to reducing movement effort. These concepts, in conjunction with other factors such as minimizing variance [38], may account for the straight-line trajectories observed in such an environment.

An ensuing question from our study is whether asymmetric effort valuation depends on the magnitude of effort. Subjects’ perceived exertion increased with resistance, but the amount of effort encountered in this task was fairly modest; reaching against even the largest resistance was not exceptionally taxing. Would we see similar degrees of gain/loss asymmetry for larger amounts of effort? In the financial domain, there is conflicting evidence regarding the extension of loss aversion to different amounts of money. Increasing availability of free-spending income has been shown to attenuate loss aversion [39], but a recent socio-demographic study found instead that higher wealth and income are associated with stronger loss aversion [40]. A reversal of loss aversion has even been observed for small monetary outcomes (e.g. less than 1€), attributed to the hedonic principle and cognitive discounting [41]. Others have shown that loss aversion increases for larger financial outcomes [39, 42], and we submit that this magnitude effect could feasibly hold for effort aversion.

It may, in fact, be difficult to extend the risky-choice lottery paradigm to more naturalistically effortful movements, as these movements could be construed as forms of exercise (i.e. walking, running, weight lifting, push-ups, pull-ups). Consider an example: would you rather run one mile for sure, or have a 50:50 chance of running either half of a mile or two miles? There are many potentially confounding factors that could affect one’s motivation and choices in a laboratory setting. Are you a frequent runner? How would this run fit in with your exercise regimen, if one exists? Are you feeling physically well and energized? Did you eat a big breakfast and want to work it off? Did you eat a big breakfast and are now lethargic? Depending on a person’s physical state and frame of mind at the time of the experiment, increased effort may not necessarily be considered a loss, and individuals may possess firm, pre-existing reference points for certain movement tasks. The possible variation in subjects’ interpretations of effort-based gains and losses complicates empirical measurement of the subjective value of effort, but it also attests to the importance of accounting for subjective value of effort to explain movement decisions. The subjects included in our study expressed a preference for lower resistances over high resistances, confirming that they viewed increased effort as a loss in the context of this experiment. Alternative methods and controls may be required to tease out relative gain/loss valuation for higher levels of effort.

We were not able to directly compare model parameter estimates between effort and money. This is due to the different value encodings of the winning models in these domains (nonlinear for EFF, linear for FIN) and the resulting effects of the parameters. Regardless, subjects exhibited similar trends of valuing losses more strongly than gains in both effort and money. Previous work has observed consistent within-subject choice behavior across domains, such as for different types of reward (i.e. food and money [43]) and different probabilities of reward (i.e. standard probability and probability based on motor performance [44], or probability based on motor performance in dissimilar movement tasks [45–47]). Our results support a more domain-specific account of loss aversion and value sensitivity, as suggested by [48]. Possible explanations may arise from a difference in familiarity between the tasks (i.e. more intuition or experience with financial outcomes), or from the relatively small level of effort examined in this experiment, which would create differences in the perceived benefits and costs between the two tasks [48].

The observed effort valuations in arm-reaching suggests that movement decisions are geared toward avoiding higher effort over acquiring lower effort. This finding has important implications for computational models of sensorimotor control. Presently, these models fail to account for subjective representations of effort that may factor into our movement decisions. Understanding subjective representations of effort will help us simulate and predict behavior for a wider range of movement decisions. Our work may also bear significance in devising exercise regimens for rehabilitation or general fitness. We have shown that imposing a reference level of effort results in asymmetric valuation of increasing and decreasing effort. Future studies may consider leveraging this asymmetry around a salient reference point to encourage healthy movement behavior, or examine the impact of financial incentives on physical effort expenditure (which has been previously studied in cognitive effort [49]).

Ultimately, our findings in arm-reaching indicate that movement decisions can be influenced by a desire to avoid higher effort over a desire to acquire lower effort. Understanding effort valuations will help us predict and incentivize behavior for a wider range of movement decisions.

## Materials and methods

### Ethics statement

All subjects provided written informed consent before participation. The experimental protocol (14–0186) was approved by the Institutional Review Board of the University of Colorado Boulder in accordance with federal regulations, university policies, and ethical standards regarding human subject research.

### Experimental protocol

During a preliminary training session, seated subjects (N = 20, 12F/8M, 23.9 ± 4.0 yrs) made horizontal planar reaching movements using a robotic handle (Shoulder-Elbow Robot 2, Interactive Motion Technologies, Cambridge, MA) while secured by a 4-point seatbelt (Fig 1A). Optical encoders sampled the position of the robot handle at 200 Hz. The position of the handle controlled a cursor (0.4 cm radius) on a computer screen in front of the subject. A single trial required the subject to move the cursor from a home circle (0.7 cm radius) to a large rectangular target (15 cm wide, 4.3 cm high) located 20 cm away from the center of the home circle. This target was designed to be notably larger than the home circle and cursor so that the task was focused on movement effort rather than endpoint accuracy. The home circle and target switched positions on every trial, so odd-numbered trials required reaches away from the body and even-numbered trials required reaches toward the body. Visual feedback encouraged subjects to complete the movement within 550–650 ms.

We manipulated the level of effort encountered during reaching by altering the damping coefficient *b* in a viscous force field:

$$F_{\mathrm{robot}}=[F_{x},F_{y}]=-b[v_{x},v_{y}]$$ (1)

This viscous field produced a resistive force *F*, opposing the direction of movement and proportional to the handle velocity *v*. Subjects first performed 100 reaches at an intermediate level of effort (*b* = 35 N⋅s/m), termed the “reference” resistance. They then trained at 10 conditions in a randomized order, including *b* values below (0, 7, 14, 21, 28 N⋅s/m) and above (42, 49, 56, 63, 70 N⋅s/m) the reference. While reaching, they were shown a quantitative level of effort to describe to the condition as a percentage of effort relative to the reference, mapped onto *b* values that were below (100, 80, 60, 40, 20% less) and above (20, 40, 60, 80, 100% more) the reference (designated as 0% less and 0% more). For each training condition, they performed 40 trials at a given resistance, followed by a 30-second rest and 20 additional trials at the reference resistance.

In the testing session, subjects were shown a series of 363 effort-based lotteries. Each lottery consisted of a 50% chance of having to reach against a higher amount of effort (above the reference, framed as a loss) and a 50% chance of having to reach against a lower level of effort (below the reference, framed as a gain). Effort lotteries were constructed across gain and loss increments of 10% (EFF, Fig 1B). Each lottery was shown three times, and the order of lotteries was randomized for each subject. Subjects indicated whether they would accept or reject each lottery using a handheld remote, and whether this was a strong or weak preference. Prior to testing, subjects were informed that a single random lottery would be selected and “played” at the end of the experiment. While playing out this lottery, subjects performed approximately 10 minutes of reaching movements (500 trials), with the same setup implemented during training. If the selected lottery had been rejected, the subject reached at the reference resistance; if the selected lottery had been accepted, a coin flip determined whether the subject reached at the higher or lower resistance.

In a separate, second testing session, 18 of the original participants repeated the effort lotteries (EFF2) and subsequently completed an analogous lottery task for financial decisions (FIN, Fig 1B). All 20 of the original subjects were invited to the second session, but two declined to participate. During this session, subjects first performed a truncated version of training, which included the reference resistance (80 trials) and four conditions in a randomized order (20 trials each, followed by 10 reference): 100% less, one of the resistances between 100% less and 0% less, one of the resistance between 0% more and 100% more, and 100% more.

The financial task involved the same 363 lotteries, shown in a different randomized order, but with the gains and losses relating to dollar amounts rather than percentage effort (FIN, Fig 1B). For this task, we endowed subjects with $30 cash at the end of the first testing session and scheduled their second session approximately one week later. The subjects brought $60 cash to the second testing session, similar to the procedures presented in [50]. We did not ask subjects whether the $60 they brought included the same $30 initially endowed to them; they were welcome to spend the endowed $30 if they so desired. One of the financial lotteries was selected and played at the end of the experiment. If the selected lottery had been rejected, the subject left with the original $60 amount; if the selected lottery had been accepted, a coin flip determined whether the subject won additional money or lost money from the $60 amount. Subjects were instructed that they would “play out” a lottery using the $60 they brought, and asked to treat each financial lottery as though it would be played. We did not select trials to play from lotteries that could result in them gaining or losing more than $30 –the amount originally endowed to them. Only a few subjects asked what would happen if they had to “play” a lottery with a loss of more than $60. If they asked, we repeated that they would only be playing using that pot of $60, and only then we explicitly stated that they would not be at risk to lose more than $60.

We chose the $30 endowment amount for reasons of financial feasibility. The sure bet (reference) does not map cleanly between the EFF and FIN tasks, since effort, as a cost, had to be translated from a pure loss to span the gain and loss domains. As such, the sure bet could not be determined identically between the two tasks, because the intermediate value of effort was still effortful (*b* = 35 N⋅s/m), but the intermediate value of money was $0 (so any loss relative to the sure bet would have resulted in taking the subjects’ own money). We selected an amount to endow that would be commensurate with the time commitment of the entire study, and subjects essentially matched that amount with their own money to maximize the feeling that they were playing with their own money rather than “house money.” Larger endowments would have been difficult given the number of subjects and the anticipated behavior of rejecting lotteries in favor of the sure bet.

### Quantifying loss aversion

We quantified loss aversion by fitting subject responses to a choice model with a curvilinear subjective value function (SV = *X*^*α*^, for *X* > 0; SV = −*λ*(−*X*)^*α*^ for *X* < 0). Gain and loss values *X* were the decreases and increases in resistance (or dollar amounts for the FIN task), ranging from -35 to 35 N⋅s/m in increments of 3.5, since these were the equivalent conditions shown to subjects during testing. The value sensitivity, *α*, describes how larger changes in a loss or gain are valued relative to smaller ones, with *α* = 1 representing objective valuation of outcomes. A parameter *α* <1 represents diminishing sensitivity, where the impact of a potential outcome decreases with distance from the reference point. The loss-aversion coefficient, *λ*, describes how losses are valued relative to gains, with *λ* = 1 representing symmetric valuation of losses and gains. A coefficient *λ*>1 traditionally represents loss-averse behavior, where losses are more undesirable than gains are desirable. Conversely, a coefficient *λ*<1 represents gain-seeking behavior, where gains are more desirable than losses are undesirable. All analyses were performed using MATLAB 2014b (The MathWorks, Inc., Natick, MA).

Maximum likelihood estimation was used to estimate subject-specific loss-aversion coefficients. The total utility of the presented lottery, with gain *X*^+^ and loss *X*^−^, is:

$$U_{Lot}=0.5X^{+}-0.5\lambda {(-X}^{-})$$ (2)

To compute the probability of accepting a given lottery, we used a logistic choice function with constant noise:

$$P_{Lot}=\frac{1}{1+exp[-\mu U_{Lot}]},$$ (3)

where *μ* is a parameter that accounts for stochasticity in a subject’s choices, and *μ* = 0 characterizes random choice. Let *r*_*i*_ be the subject’s choice on the *i*th trial, with *r*_*i*_ = 1 denoting acceptance of the lottery and *r*_*i*_ = 0 denoting rejection of the lottery in favor of reaching against the reference resistance. The estimated parameters (*λ*, *α*, *μ*) maximize the likelihood function over *n* trials:

$$L(\lambda ,\alpha ,\mu )=\prod_{i=1}^{n}P_{Lot}^{r_{i}}{\left(1-P_{Lot}\right)}^{r_{i}},$$ (4)

We used the MATLAB routine *fminsearch* with multiple restarts to minimize the negative likelihood function, thereby finding the maximum likelihood estimate for this model.

Alternative models, including exploration of nested value functions and effort representation, were fit in the same way, with the exception of the 3-parameter value function. Unconstrained optimization of the 3-parameter model resulted in a number of parameter fits outside the scope of prospect theory (i.e. negative sensitivity, negative loss aversion coefficient). We thus applied constrained optimization (routine *fmincon* in MATLAB) for this model, setting the lower bound of each parameter to 0 and the upper bound to 10 for *α*, *β*, *λ* and 20 for *μ*.

### Ratings of perceived exertion

We also asked participants to rate their perceived exertion for different resistances to assess how their perception of effort scaled with viscous force and whether the changes in effort we presented were discernible to them. Prior to the training session, subjects performed reaches for the aforementioned training conditions without receiving feedback about the levels of effort. Instead, subjects themselves evaluated the amount of effort required to complete a movement using Borg’s Rating of Perceived Exertion scale [51]. During this portion of the experiment, subjects were exposed to the lowest effort condition then the highest effort conditions, then the remaining conditions in a randomized order. For each condition, they performed 50 trials at a given resistance, followed by a 30-second rest during which they gave a rating of perceived exertion for that resistance and 20 subsequent washout trials with no resistance. They were notified in advance when performing the lowest and highest effort conditions, and were asked to consider this when determining their level of exertion for those conditions and the remaining conditions. In doing so, we intended that their exertion ratings would cover a broad range on the Borg scale to help subjects verbalize perceived differences between the 11 effort conditions.

Subjects additionally filled out an exit questionnaire in which they ranked the training conditions (from 100% less effort to 100% more effort) in order of their preferences if they had to perform 10 minutes of reaching against some resistance. The purpose of this questionnaire was to ensure that participants indeed perceived increasing effort as a loss. We excluded any subjects from our study if (1) they indicated a reversal of this preference on the questionnaire, or (2) they did not demonstrate the expected monotonic trends in lottery rejection (increased rejection of increasing losses and decreased rejection of increasing gains). Two additional subjects were tested in the first EFF session who exhibited such behavior and have not been included in our analysis.

### Alternative models

We explored various alternative models to explain subject choice behavior. We compared the 3-parameter subjective value model:

$$SV=X^{\alpha },\;\mathrm{for}\;X>0$$

$$SV={-\lambda (-X)}^{\alpha },\;\mathrm{for}\;X<0$$

where *λ*, *α*, and *μ* as free parameters, to a nested 2-parameter model without value sensitivity (linear value function, with *α* = 1, and *λ*, *μ* as free parameters) and a nested 1-parameter model without sensitivity or loss aversion (*α* = *λ* = 1, and *μ* as a free parameter). Inspired by previous results suggesting a quadratic encoding of effort, these models were tested using both linear and quadratic representations of the effort lotteries (Fig 7B). We fixed the exponent, *γ*, on the damping coefficient, *b*, to be one (linear) or two (quadratic):

X=Δ(b)

$$X=\Delta (b^{2})$$

This means that the underlying effort representation is first transformed (either linearly or quadratically), and then the difference is calculated with respect to the transformed reference value to obtain the resulting gain or loss for each lottery.

To determine the effectiveness of the artificially imposed reference point (*b* = 35 N⋅s/m), we also considered a model with a zero-effort reference point, in which all effort outcomes would be considered losses. Here, subject choices were fit to a curvilinear value function in the loss domain with sensitivity *γ*:

$$X=b^{\gamma }$$

Lastly, we also considered a hybrid model combining the reference-dependent loss aversion, *λ*, present in traditional prospect theory models, with an underlying nonlinear encoding of the effort function, *b*^*γ*^:

$$X=\Delta (b^{\gamma })$$

SV=X,forX>0

SV=−λ(−X),forX<0

Here we fit both the loss aversion parameter, *λ* and the effort encoding parameter, *γ*, in contrast to the previous models where *γ* was explicitly fixed to be linear or quadratic. For this hybrid model we fixed relative effort sensitivity, *α* = 1. When we allow for nonlinearity in absolute effort sensitivity, *γ*, this means that combinations of *λ* and *α* can provide non-unique manifestations of loss aversion. Thus to provide a unique representation of asymmetric valuation of losses relative to gains, we constrained *α* and fit only the loss aversion parameter, *λ*.

A similar model space was tested for the FIN task, including a large-money reference model in which all monetary outcomes were framed as gains via linear translation. The quadratic valuation was not tested in the FIN task, resulting in 5 total models. All models were run with multiple restarts to determine the maximum likelihood estimate.

For each task, the most likely model was determined via Bayesian model selection [52, 53], a group-level random effect analysis. Posterior model frequencies, protected exceedance probabilities, and Bayesian omnibus risk (*P*_*0*_) were computed from the marginal likelihoods of subjects’ parameter fits, computed via the Aikaike Information Criterion, using Statistical Parametric Mapping software (SPM5, Wellcome Trust Centre for Neuroimaging). The protected exceedance probability is the probability that a model is more frequent than others in the competing model space, against the null hypothesis that all models in the space are equally frequent. The winning model were selected as that with the highest protected exceedance probability. In all tests, this value was above the recommended disambiguation threshold for our relative sample size [53]. Bayesian omnibus risk is the probability that the observed difference in model frequencies may have occurred by chance. Model identifiability was confirmed with a confusion analysis [54], wherein lottery choices were simulated for 20 subjects in each potential model from the prior distribution of parameters, then each candidate model was tested on the simulated data and Bayesian model selection was performed on the resulting parameter estimates. The process was repeated for eight Monte Carlo simulations, testing for confusion in recovering each model. Additional Monte Carlo simulations were not considered due to consistency of model selection in each simulation.

A two-way repeated measures ANOVA was used to compare subjective valuations of gains and losses for the winning model in each task, specifically testing for effects of domain (gain vs. loss), effort condition, and their interaction.

### Statistics

A Mann-Kendall test assessed the monotonicity of mean frequency of lottery rejection across task gains and losses. We used paired t-tests to examine differences in frequency of lottery rejection between the EFF and FIN tasks across gains and losses, adjusting for 11 multiple comparisons using the Bonferroni method. Normality of all parameter fits was assessed using the Shapiro-Wilk test, and Wilcoxon signed-rank tests were used to compare parameters to unity or between tasks when normality was rejected. We computed Pearson’s correlation coefficient to compare estimates of *λ* and *γ* between the EFF and EFF2 tasks. Unless otherwise specified, the significance level was set to 0.05.
